# Quartz crystal microbalance and atomic force microscopy to characterize mimetic systems based on supported lipids bilayer

**DOI:** 10.3389/fmolb.2022.935376

**Published:** 2022-08-03

**Authors:** Noel F. Bonet, Daniel G. Cava, Marisela Vélez

**Affiliations:** Instituto de Catálisis y Petroleoquímica (CSIC), Cantoblanco, Madrid, Spain

**Keywords:** atomic force microscopy, quartz crystal microbalance, supported lipid bilayers, biomimetic membranes, label-free detection

## Abstract

Quartz Crystal Microbalance (QCM) with dissipation and Atomic Force Microscopy (AFM) are two characterization techniques that allow describing processes taking place at solid-liquid interfaces. Both are label-free and, when used in combination, provide kinetic, thermodynamic and structural information at the nanometer scale of events taking place at surfaces. Here we describe the basic operation principles of both techniques, addressing a non-specialized audience, and provide some examples of their use for describing biological events taking place at supported lipid bilayers (SLBs). The aim is to illustrate current strengths and limitations of the techniques and to show their potential as biophysical characterization techniques.

## Introduction

Biological membranes are essential for life. They play a central role in cellular structural integrity, signaling pathways, chemical compartmentalization, and are constituted primarily by phospholipids and integral and peripheral proteins. Studying interactions between lipids and proteins underlying complex functions in real cells is very difficult to approach experimentally and results are in many instances challenging to interpret unequivocally. This circumstance has triggered efforts over the years to develop simpler mimetic systems that facilitate addressing quantitative and important issues related to such aspects as the physical-chemistry of lipid interactions, their spatial distributions, phase segregations, or how their presence modulates membrane associated proteins and their interaction with other proteins or with soluble ligands (Andersson et al., 2020). This issue dedicated to “Biomimetic and Bioinspired Membranes to Reconstruct the Properties of Natural Systems” includes articles that illustrate the use of biomimetic systems and their contribution to advancements in different fields. This article focuses on presenting two experimental techniques, Quartz Crystal Microbalance and Atomic Force Microscopy, used to characterize processes occurring at air-solid or liquid-solid interfaces and that can also be used in combination to provide complementary information about events taking place on supported lipid bilayers, one commonly used biomembrane model system.

There are mainly two mimetic experimental platforms used to study physicochemical properties of biological membranes and protein lipid interactions: artificial lipid vesicles and supported lipid bilayers (SLBs) (Sezgin and Schwille, 2012), (Céspedes et al., 2021). Unilamellar lipid vesicles with diameters that can range from a few tens of nanometers, (referred to as Small Unilamellar Vesicles, SUVs, and Large Unilamellar Vesicles, LUVS) (Rhoden and Goldin, 1979) to up to several microns (Giant Unilamellar Vesicles, GUVS) can be prepared using different protocols (Angelova and Dimitrov, 1986), (Staneva et al., 2021). GUVs have received much attention in the last decades as they are large enough to be studied using optical microscopy and their diameter is comparable to that of cells. In lipid vesicles the membranes are surrounded by solution on both sides. These “free standing” membranes therefore closely mimic frequent real cell conditions and avoid worries about potential adverse interactions with the substrate. In contrast, SLBs deposited on a solid substrate are flat and extend over many millimeters, which has the advantage of permitting the use of a battery of characterization tools, like the ones described in this work, to obtain time resolved structural descriptions of the evolution of processes taking place at the interface. In addition, in some cases cell membranes are closely associated with proteins that sit at either side, such as cytoskeletal or extracellular membrane proteins, whose association affects the behavior of the lipids on the membrane. In such instances, supported membranes provide a mean to reproduce and control experimentally events that take place under such functionally relevant situations. Furthermore, fluid lipid membranes with functional proteins incorporated on surfaces are also required for bio-sensing applications or to create controlled tailored cell substrates to modulate cell attachment, growth or differentiation, either for basic studies or for biotechnological applications (van Weerd et al., 2015).

SLBs were first developed in the mid 80’s and since then, different preparation and characterization methods have been developed**.** The first report described the transfer of lipid monolayers formed in a Langmuir trough to solid supports and characterized using fluorescence microscopy (Tamm and McConnell, 1985). The choice of preparation protocol depends on the techniques to be used and the purpose of the studies. The Langmuir-Blodgett/Langmuir-Schäfer (LB/LS) methods are convenient for the preparation of asymmetric bilayers. However, the most common technique, developed shortly after the original Langmuir based methods, is direct vesicle fusion (Watts et al., 1984), (McConnell et al., 1986). More recently, microfluidics has also been employed for their preparation (Janshoff and Steinem, 2007), (Reviakine et al., 2005). The motivation to enrich the complexity of SLBs and to include integral membrane properties triggered the need to devise strategies to lift the supported membranes from their solid supports by the introduction of an intervening polymer cushion that improves the lateral mobility of integral membrane proteins (Veneziano et al., 2017), (Clifton et al., 2020), (Andersson and Köper, 2016), (Goennenwein et al., 2003), (Wagner and Tamm, 2000). The use of polymer cushions can also be advantageous to preserve symmetric and asymmetric lipid domain structures in supported membranes.

**FIGURE 1:**
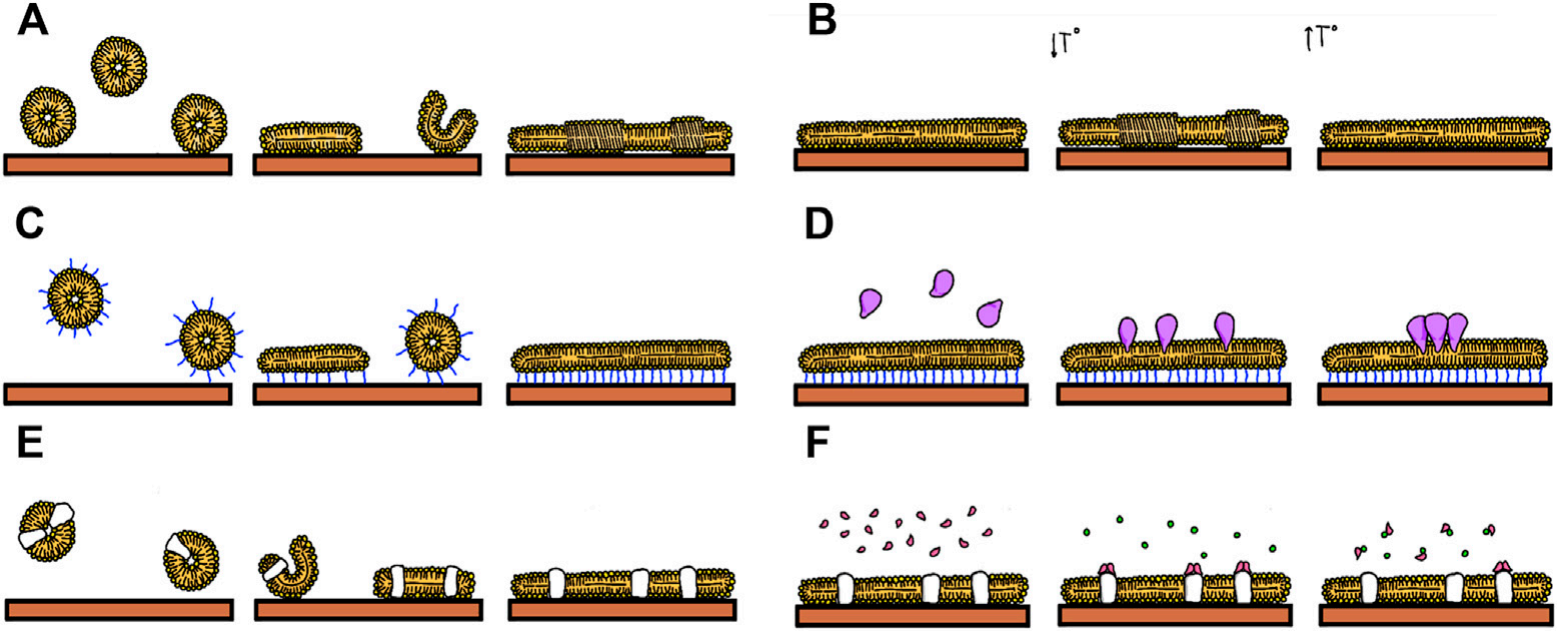
Events that can be studied using Supported Lipids Bilayers: **(A)** Formation by vesicle fusion. **(B)** Formation of lipid domains **(C)** Formation of tethered bilayers **(D)** Insertion of proteins **(E)** Fusion of proteoliposomes **(F)** Following biomolecular interactions such as that between soluble ligands and membrane proteins.

There are recent excellent reviews describing protocols to prepare SLBs with and without proteins and the reader is referred to them for a practical guide for their preparation (Andersson et al., 2020), (Kiessling et al., 2015), (Andersson and Köper, 2016). A quantitative analysis of several structural and dynamic aspects of the evolution of the mimetic systems is required to extract relevant information. Initially, we will first wonder how the lipid bilayer spreads over the solid support. We might be interested in optimizing the speed and extent of coverage as a function of the material we are interested in covering, its roughness or incubation conditions. Examination of the process as a function of the type of lipids with varying charges and transition temperatures can also be of interest, as well as characterizing the state of the lipids on the covered surface. If membrane associated proteins are the subject of study, it might be of interest to quantify their incorporation, how they distribute on the surface or how they interact with soluble ligands (Figure 1).

The two techniques described here, Quartz Crystal Microbalance and Atomic Force Microscopy are well suited to study hydrated soft material deposited at the solid liquid interface and can therefore be used to address some of these questions. Both are excellent tools to characterize interfaces without requiring any type of sample labeling, in contrast to the widely used surface characterization tools based on fluorescence. QCM can detect the amount of mass deposited on a solid surface and the AFM can follow the profile of material deposited with enough sensitivity to distinguish height differences of less than one nanometer, providing then complementary information about surface coverage and how the material is deposited and distributed on the surface. The QCM is able to monitor changes in the resonance frequency of a quartz crystal due to the presence of mass on the surface. Typically, a 5 MHz quartz sensor is able to detect as little as 18 ng cm^−2^. The data acquisition in both techniques is done with the surface immersed in solution. QCM can provide real time measurements of the amount of mass that deposits, and a parallel characterization of the surface using AFM can describe the homogeneity and thickness of the material distribution. Recent technical and theoretical developments directed towards combining them with additional detection systems, increasing their time and space resolution and refining the theoretical analysis to include how the morphology and disposition of the hydrated material affect the resonating quartz crystal will most surely open the door to obtain new and more precise information about how the lipids and proteins are displayed on the surface. The aim of this work is to describe the fundamental operation principles of both techniques to a non-specialized audience in order to emphasize their current potential as biophysical characterization techniques and to outline current applications. Some examples will be provided to illustrate current strengths and limitations of the techniques.

## Quartz Crystal Microbalance

### Basic principle

**FIGURE 2:**
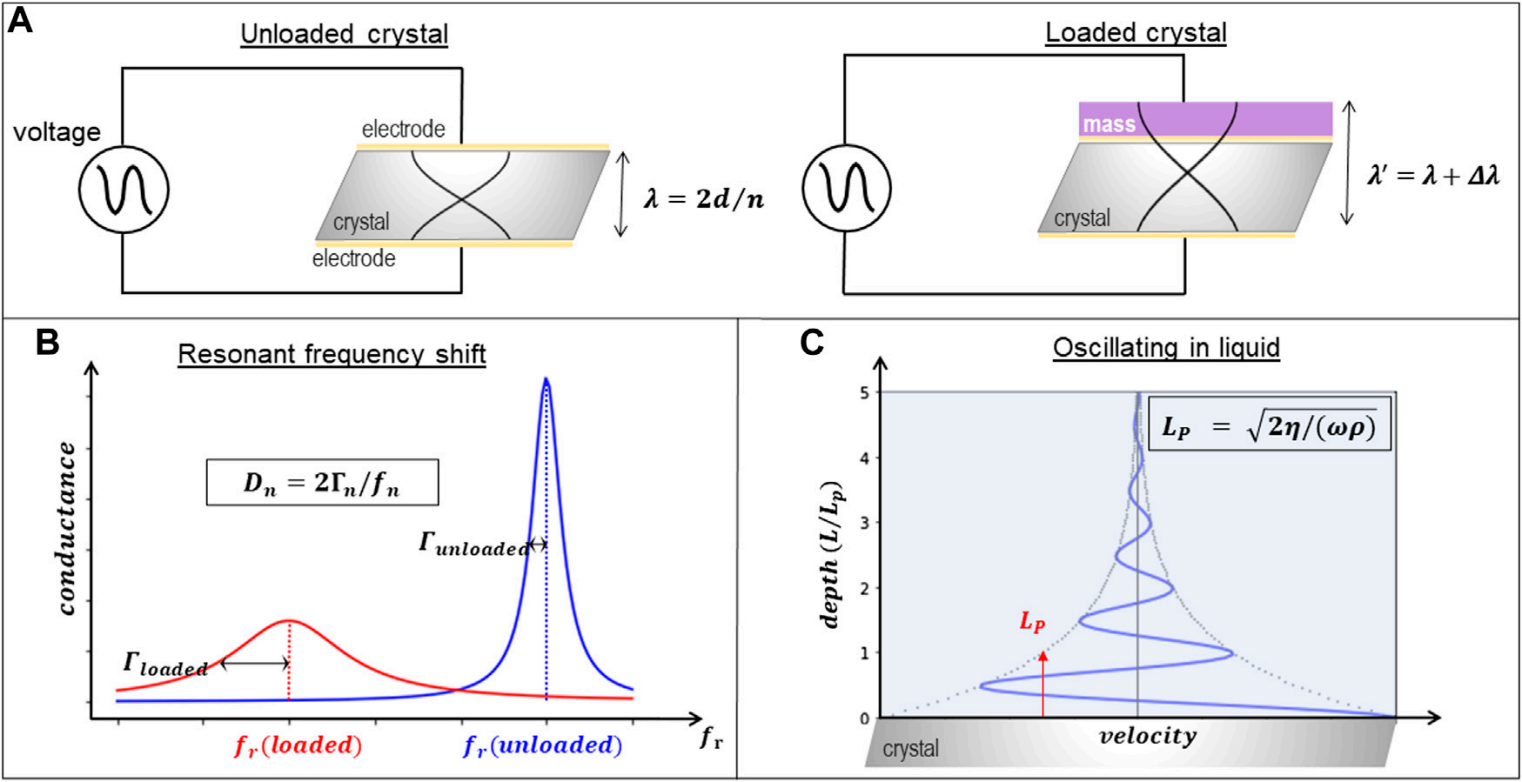
Description of QCM operation principle. **(A)** Oscillation of the quartz crystal (of thickness d) driven by the electrodes without (left) and with (right) material deposited on top illustrating how the wavelength λ of the resonance frequency changes to λ ’ for different overtones n. **(B)** Illustration of the energy dissipation (D_n_) extracted from the bandwith (Γ_n_) and frequency (f_n_) changes of the loaded crystal. **(C)** Illustration of the penetration length (L_p_) that depends on the frequency (ω), density (ρ) and viscosity (η) of the solution.

A QCM is an acoustic device that detects the deposition of material on the surface of a resonating quartz crystal that acts as a piezoelectric transducer. It can operate in air or immersed in solution. Due to its low operating cost, sensor compactness, real-time data, label-free operation and subnanogram sensitivity, it has become a fundamental tool in analytical chemistry and biophysics research. QCMs are simple and versatile devices that consist of thin disks made from a piezoelectric quartz crystal, cut in the AT angle, sandwiched between two metal electrodes that establish an alternating electric field across the crystal at its resonant frequency, which is sensitive to mass changes of the crystal and its electrodes. When the instrument operates in the thickness shear mode, as happens in most commercially available QCMs, the two crystal surfaces undergo a lateral oscillatory antiparallel displacement. The vibration amplitude and penetration depth of the oscillation strongly depend on the resonance frequency of the crystal and the viscosity of the bulk solution. For a 5 MHz crystal, the amplitude of vibration is usually 10 ± 20 nm in air and is reduced in water to 1 ± 2 nm. This oscillation then produces a lateral shear wave that penetrates into the bulk solution around 250 nm before being attenuated. In resonance, crystal surfaces are located at the antinodes of a standing wave with the wavelength 2d/n, where d is the crystal thickness and n is the overtone order (odd), leading to the resonance frequency f_n_ = nc/2d, where c is the speed of sound in quartz (Reviakine et al., 2011). In liquids and gases, shear-waves decay rapidly, making QCM interface-specific, as illustrated in Figure 2.

The basic principle underlying the mass measurement is the existence of a linear relationship between deposited mass and the frequency response, as demonstrated by Sauerbrey (1959). Sauerbrey relation is only strictly valid in vacuum, yet it is commonly used in liquid QCM, provided that the adsorbed mass is small compared to the weight of the crystal and that it is rigidly adsorbed and evenly distributed over the active area of the crystal. These circumstances are seldom true when looking at proteins deposited on lipid bilayers, as will be further described below. The linear relation between changes in frequency, Δf, and adsorbed mass, Δm, is therefore often referred to as the Sauerbrey relation (Sauerbrey, 1959):

$$\Delta m=-C\frac{\Delta f}{n}\;$$

Where Δ*f* is the measured frequency change (Hz), Δ*m* the change in mass (mass per unit area) and n is the overtone order (odd). The mass sensitivity constant C depends on the fundamental frequency of the quartz crystal prior to a mass change, the electrode area and the density shear modulus of quartz. Typically, for a 5 MHz commercially available crystal C = 17.7 ng cm^−2^ Hz^−1^. This extremely small limit of detection has contributed to the extended use of this technique as a mass balance.

This sensing principle, schematically summarized in Figure 2, was originally utilized almost exclusively for gas-phase and vacuum applications, the most common use being as a thickness monitor during film depositions in vacuum systems where perturbations such as mechanical vibrations, stress, or temperature gradients are controlled. Under these conditions, the technique has enough sensitivity to detect much less than a monolayer of gas molecules adsorbing or desorbing from the surface.

**FIGURE 3:**
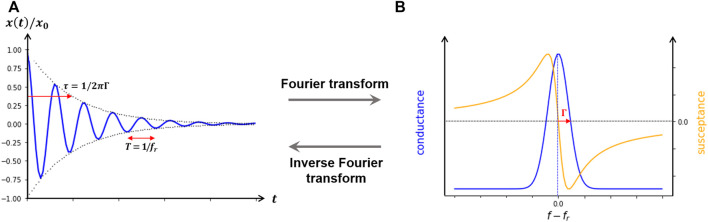
Modes of detecting resonant phenomena. **(A)**, ring-down method, where the driving signal is abruptly shut off the decay as a current trace on an oscilloscope is observed. **(B)**, impedance analysis in which one may either sweep the frequency of an AC excitation across the resonance.

**FIGURE 4:**
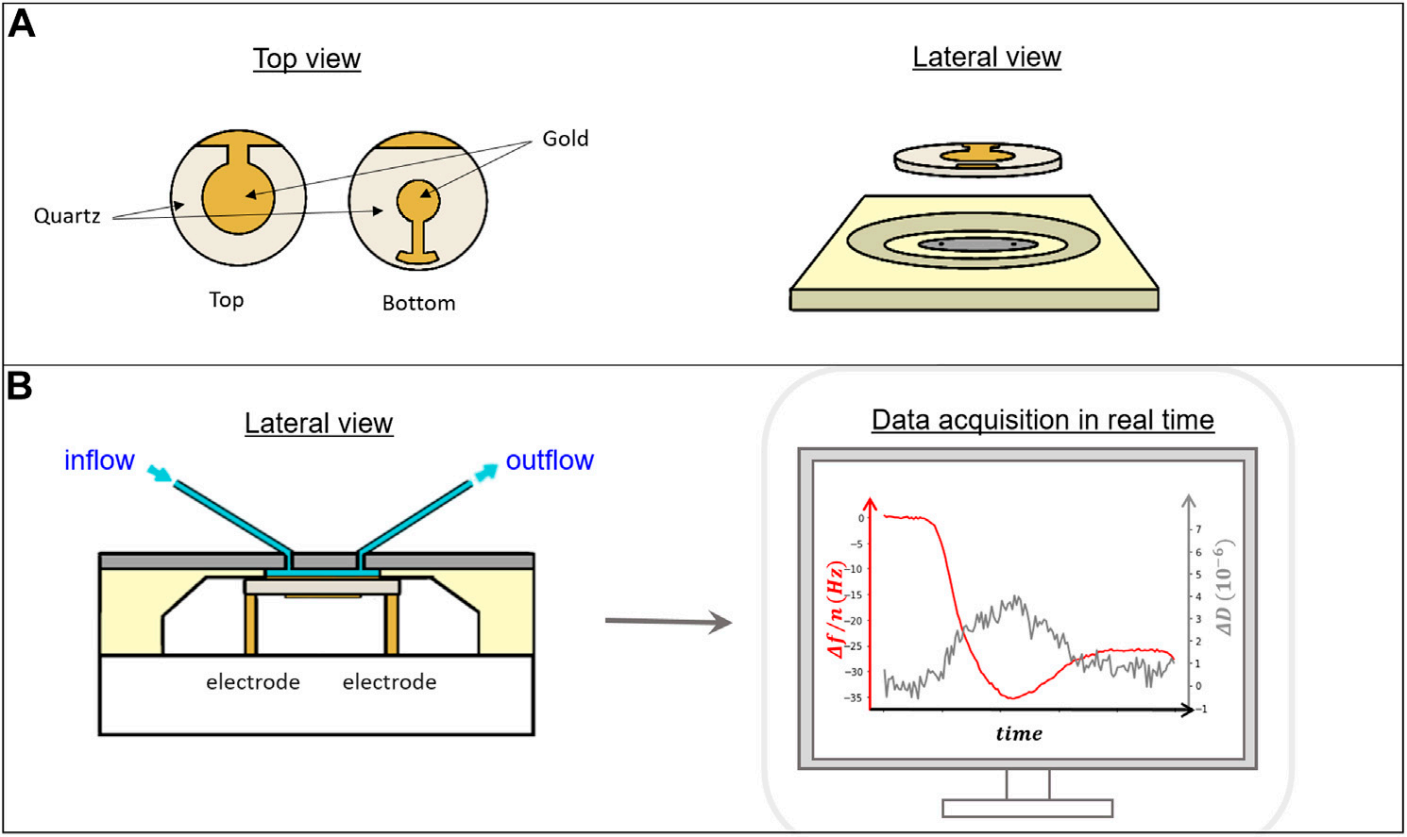
**(A)** Top and side view of a quartz crystal showing the gold electrodes on top and bottom that allow the connection to electrically drive the crystal oscillation. **(B)** Lateral view of a diagram of the QCM measuring chamber (left). The crystal is inserted into a sealed chamber that allows contact with the electrodes from the bottom and exposure of the active upper region to liquid that can be exchanged through a pumping system. Right, example of a typical data acquisition set where the frequency drop (red line) registered follows the adsorption of material to the crystal surface and the dissipation in real time (gray line) (Figure 5 for further details).

Detection of the changes in frequency due to alterations in the mass of the crystal during the development of the experiment requires following its resonant oscillation over time. Resonant phenomena can be probed in either the frequency domain or the time domain. The two modes of interrogation yield equivalent information, as long as the dynamical equations are linear. One may either sweep the frequency of an AC excitation across the resonance, as in impedance analysis, or abruptly shut off the driving signal and watch the decay as a current trace on an oscilloscope, as in ring-down (Figure 3). In both cases, there are two parameters detected per overtone, the resonance frequency f_n_, associated to the amount of mass deposited on the surface, and the dissipation D_n_, associated to the loss of energy of the oscillation as the material is deposited. In the impedance analysis, the bandwidth Γ_n_ (shown in Figures 2B, 3B) reflects the dissipation. The alternative “ring-down” scheme was developed by Rodahl et al. (1995), and implemented in the instrument marketed by Biolin Scientific (Västra Frölunda, Sweden) (Johannsmann et al., 2021), and is referred to as QCM-D. The external driving voltage is turned off intermittently and the oscillations are left to decay freely. Given that quartz is piezoelectric, the voltage generated during these decaying mechanical oscillations is recorded, also yielding two parameters per overtone, the resonance frequency f_n_ and the dissipation D_n_ (Figure 3A). Bandwidth and dissipation are equivalent, with D_n_ = 2Γ_n_/f_n_ and both terms can be used. The third way to perform QCM measurements is with oscillator circuits. These can be very economic but usually operate on one harmonic only and provide only indirect access to the bandwidth via the oscillation amplitude. This limits the data interpretation severely. All of these approaches require that the quartz crystal is coated with electrodes, as is illustrated in Figure 4.

Operation of the QCM acoustic device is relatively simple. The crystal is inserted in a close chamber (Figure 4) and the solution containing the biological sample can flow through the chamber fed through a controlled pumping system. The typical operating volume within the chamber is around one hundred microliters, so the amount of material required for each experiment is small. Solutions contain typically micromolar concentrations and, depending on the time required for the reactions of interest to occur, the total volume of solution required for each experiment is around a few mililiters. The crystal surface is typically covered by gold, that acts as the electrode to drive the oscillation of the crystal. As gold is hydrophobic and does not support vesicle fusion unless modified, there are commercially available crystals in which a thin film of other materials such as SiO_2_, better suited to form SLBs from vesicle fusion, is deposited on top without interfering with the crystal oscillation. Some manufacturers provide commercially available crystals covered with other materials and offer the possibility of modifying the surfaces to adapt to costumer needs.

The lateral shear wave generated by the crystal oscillation penetrates into the bulk solution around 250 nm for a 5 MHz crystal in water, as was mentioned above. This length, usually called penetration depth, depends on the characteristics of the crystal and the solution, and decreases as the square root of the working frequency (Figure 2C). Events that affect energy dissipation occurring within this spatial region above the surface can also be sensed in the measurement. Changes in the viscoelastic properties of surface bound or surface proximal materials such as polymer films, their roughness, surface charge density, the water content of biomolecules or morphological changes that alter their hydrodynamic behavior at the interface are all phenomena that dissipate energy (Janshoff and Steinem, 2007). Therefore, measuring in real time the changes in resonance frequency Δf and in energy dissipation, ΔD, during an interfacial process can provide information both about changes in the amount of mass, and the disposition or the viscoelastic properties of the material on the surface. However, extracting and interpreting the information provided by these changes is complex. It requires a hydrodynamic analysis coupling the adsorbed material properties with the fluid dynamics around the adsorption sites that give rise to the detected signals.

### Data interpretation

**FIGURE 5:**
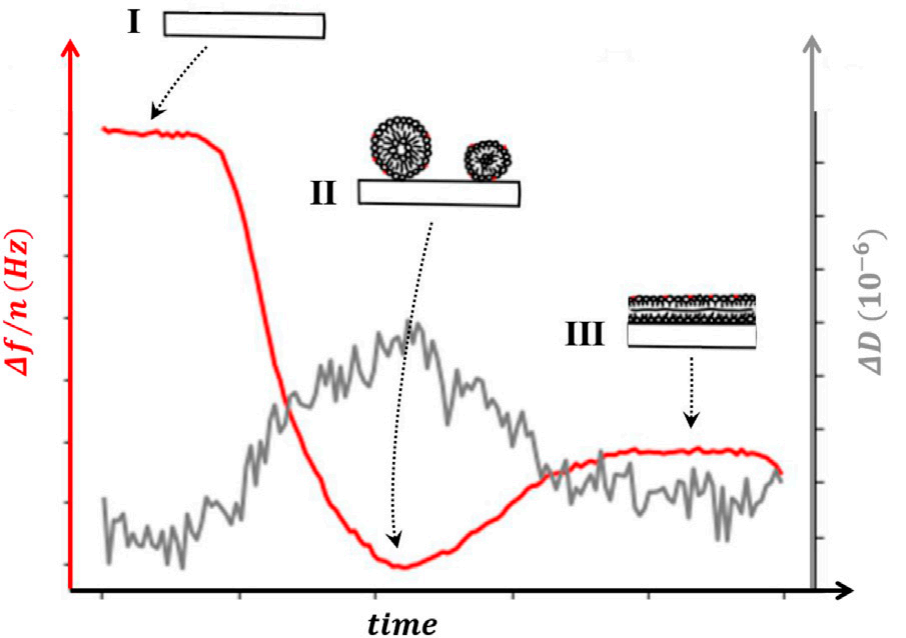
Sensogram. The change in frequency Δf/n (left axis) and dissipation ΔD (right axis) as a supported lipid bilayer (SLB) is formed from vesicle fusion. The regions depicted (I, II and III) illustrate the configurations of the sample that can account for the changes observed in sensogram signal. (I) the chamber is filled with the desired buffer and frequency and dissipation shifts are taken as reference for the rest of the experiment. (II) intact liposomes adsorbed on the surface shift both Δf/n and ΔD. (III) liposome rupture releases water, reducing both the frequency change and the dissipation, as the lipids spread on the surface to form the SLB.

Analytical QCM models have been developed to explain a simple situation in which the adsorbed layer forms an homogeneous thin-film (Sauerbrey, 1959), (Reviakine et al., 2005). Viscoelasticity is well understood for planar layer systems (Lucklum et al., 1999), (Johannsmann, 1999), so in such condition, the models allow extracting the mass and viscoelastic properties of the film. However, the study of biological materials is frequently far from being properly depicted as a set of one or more slabs with certain thicknesses. Adsorption of soft biological samples more frequently proceeds through the formation of inhomogeneous layers at intermediate coverages (Gillissen et al., 2018) of discrete sized particles, proteins, liposomes or viral capsids, whose height can protrude significantly into the solution distances comparable with the penetration depth of the shear wave. According to a commonly used interpretation, the combined effect of hydration water and/or water trapped between adsorbed species, and the non-rigid character of many biomolecules, induces frictional (viscous) losses in the deposited sample film that damp the crystal’s oscillation. When this damping becomes sufficiently large, the simple linear relation between Δf and Δm described in the Sauerbrey equation is no longer valid. In such inhomogeneous layers, even if the dissipation is low, the relation does not hold. However, these apparent limitations also represent a potential advantage of QCM to study biological interfacial phenomena, as it can provide information about both, the amount of material adsorbed and its organization at the interface. Other optical techniques such as Surface Plasmon Resonance or ellipsometry, cannot distinguish a layer of adsorbed liposomes from a flat lipid bilayer, which is, in most cases, possible from QCM raw data (Cho et al., 2010) (Figure 5). QCM with dissipation data can distinguish smooth layers of monomeric protein from piles of aggregates (Johannsmann et al., 2008), monomeric and clustered membrane-bound proteins, (Carton et al., 2010), straight, kinked, and looped surface-grafted DNA molecules (Tsortos et al., 2008a) and so on, which constitutes a strong incentive for using this technique.

Extracting quantitative information from the experimental observables, Δf and ΔD, requires interpreting these changes according to a model that explains how these parameters are associated to the mechanical and hydrodynamic phenomena occurring at the interface. There has been considerable effort in the past years to develop theories and analyses to interpret the changes in the dissipation observed as biological materials adsorb (Rodahl et al., 1997). The challenge is to assign the changes in Δf and ΔD to a physical phenomenon: amount of protein adsorbed to the surface, differences in which the protein interacts with the surface (slippage), changes in surface roughness, in protein hydration state, protein lateral aggregation or intrinsic viscosity due to conformation changes. Moreover, it has been confirmed that even if the film is firmly attached to the surface and does not present significant dissipation, the Sauerbrey approximation does not hold. Experiments in which the amount of material was measured using an independent technique, an AFM coupled with the acoustic sensor in this case (Johannsmann et al., 2008), the relation between coverage and frequency shift was found to be nonlinear, showing that ∆f is not proportional to surface coverage, even if ∆D was much smaller than |∆f| and the overtone dependence of the normalized frequency shift, ∆f/n, was small, conditions that are sometimes considered to be enough to allow the Sauerbrey approximation.

For large enough values of ΔD, predictions made by the analysis as a homogeneous film, using its acoustic thickness and shear elastic moduli as fitting parameters, do not hold either. For a homogeneous film, the ratio between the dissipation and frequency shifts, ΔD/Δf (the acoustic ratio), does not increase with surface coverage. However, in heterogeneous films, such as those composed of adsorbed colloidal particles, this ratio increases with increasing particle size but decreases with increasing surface coverage, confirming that their QCM response does not conform with the predictions of the homogeneous film model (Tellechea et al., 2009).

The essence of the problem of extrapolating the predictions for the homogeneous film to heterogeneous discrete biological samples lies on the fact that the frequency shift depends linearly on stress pattern at the interface between the resonators and the sample, according to the small-load approximation used to interpret the QCM signals. This approximation, which assumes the condition that Δf/f << 1, valid for most experiments, relates shifts of frequency and bandwidth with the stress pattern at the boundary between the resonators and the adsorbed sample (Johannsmann, 2008). In a viscoelastic homogeneous sample, the stress to the surface is completely carried by the adsorbed film, which permits to clearly distinguish different types of “loads”: the inertial load (or “mass load”) and those coming from the elastic and viscous response of the sample. However, for structured and inhomogeneous (discrete) samples the liquid phase is also in direct contact with the surface, and there is not a clear-cut way for these interpretations. In fact, most of the surface stress is due to the embedding fluid, which propagates the inertia and elasticity of the samples encoded in a complicated (viscous) hydrodynamic way. For this reason, when dealing with heterogeneous samples, it is not straightforward to associate ∆f to mass and ∆D to viscosity. Ideally, the information carried out by ∆f and ∆D should be decrypted by analyzing the hydrodynamic fields.

To advance the field and develop the full applications of this technique two complementary developments have been followed: combining measurements with other mass detection techniques and refining theoretical analyses. QCM instruments have been coupled with other surface techniques that can also detect the mass on the crystal surface. This can be done optically by monitoring differences in the optical density between the layer of adsorbed material adjacent to the surface and bulk solution. Surface plasmon resonance, waveguide spectroscopy, ellipsometry, or reflectometry have been used (Hook et al., 2002), (Vörös, 2004), (Carton et al., 2010). An alternative is to detect the volume of adsorbed material directly using microscopy techniques such as AFM (Johannsmann et al., 2008). Combining the data of the amount of mass detected by the QCM, which includes the water content, with those obtained from another technique that detects different physical properties of the mass can be helpful to discriminate the QCM-signal coming from the biological material and the one contributed by the hydration layer. If models are available, this information can be interpreted in terms of how the mass is distributed or displayed on the surface (Carton et al., 2010).

One of the models proposed to analyze QCM data quantitatively without the need of additional information provided from other techniques is to use the ratio ∆D/∆f to measure of the intrinsic viscosity [η] of the attached molecules (Harding, 1987). This ratio is frequently employed in acoustic analysis and represents the change in measured energy dissipation per surface-coupled unit mass. It has been considered to be a qualitative fingerprint of the studied bio-layer. Intrinsic viscosity [η] is a hydrodynamic property directly related to the hydrodynamic volume/shape of the biomolecule in solution (Tsortos et al., 2008a), (Melzak et al., 2009), (Tsortos et al., 2011), (Milioni et al., 2017). The assumption is that attached biomolecules behave as discrete particles instead of a film. The model relates the intrinsic viscosity [η] mathematically to the geometrical features of the biomolecule under study, i.e., its shape and size, by using well known equations derived for polymers. This approach allowed discriminating surface attached double stranded (ds) DNA molecules of various sizes but same shape or DNAs of same size but various shapes (rod, bent, triangle) (Tsortos et al., 2008b), (Tsortos et al., 2008a), (Tsortos et al., 2016), (Tsortos et al., 2016), (Tsortos et al., 2008a) and also different compaction states of protein associated to the membrane through an intrinsically disordered region (Mateos-Gil et al., 2016).

Another approach to estimate the dry mass of adsorbates in a liquid environment only from QCM measurements was recently suggested (Armanious et al., 2021). The authors showed that the liquid contribution for rigid systems can be eliminated by measuring the response in solutions with identical kinematic viscosity (ratio between viscosity and density) but different densities, which can be achieved simply by exchanging the liquid medium. The validity of this assumption-free mass quantification was demonstrated with experiments to quantify the dry mass of different materials using the kinematic-viscosity matching and comparing it to mass determined using complementary methods.

As mentioned above, a thorough theoretically explanation of what happens in heterogeneous discrete biological samples requires computing the distribution of tangential stress on the surface. To circumvent this complex challenge, an alternative phenomenological approach was proposed (Bingen et al., 2008), which reproduces the observed frequency shifts by introducing an extra mass of trapped solvent into the Sauerbrey relation with a specific confined geometry*,* namely, assigns a pyramid-shaped hydration coat to each adsorbed particle attached to the oscillating surface. This approach is not amenable to a rigorous analytical description, but it provides a useful route for comparison between systems from the estimation of the amount of trapped solvent on the surface. Deviations from the Sauerbrey relation in terms of frequency, are then translated into differences in deposited mass, which is exactly what you can obtain by combining two independent measurements of the amount of mass, as described above. The trapped solvent model permits to interpret the data and allow extracting information regarding the aggregation state (Carton et al., 2010) and size of the adsorbed particles (Tellechea et al., 2009), (Bingen et al., 2008).

The relevant role of hydrodynamics in determining the observed signals was soon recognized in the field. Pioneer numerical approaches to solve this complicated unsteady flow problem were based on finite element methods (FEM) in two dimensions (Johannsmann et al., 2008), (Carton et al., 2010) and later in three dimensions, using the lattice Boltzmann model (Johannsmann and Brenner, 2015) (Gillissen et al., 2018). These works confirmed the extreme relevance of the solvent hydrodynamics in determining QCM signals of discrete samples. More recently, other theoretical approaches have been proposed to partially decrypt the complex interplay between the solvent flow and the viscoelastic nature of the analyte (e.g. liposomes, biomolecules, etc.) (Vázquez-Quesada et al., 2020), (Meléndez et al., 2020), (Schofield and Delgado-Buscalioni, 2021). Analytical expressions for the acoustic impedance require dealing with unsteady low Reynolds hydrodynamics (Schofield and Delgado-Buscalioni, 2021), (Fouxon and Leshansky, 2018). Interestingly, the hydrodynamic approach is not based on an estimation of the water content trapped by the adsorbed molecules. On the contrary, hydrodynamic theory (Meléndez et al., 2020), (Schofield and Delgado-Buscalioni, 2021) predicts that a large contribution to ∆D and ∆f results from the motion of the solvent induced by the analyte’s presence. Novel computational approaches to QCM hydrodynamics (Vázquez-Quesada et al., 2020), do not need to fix the particle position nor to assume a particular geometry, and are able to introduce the effect of particle elasticity.

The model shows that analyte-induced hydrodynamics is usually the leading source of acoustic impedance in QCM with dissipation. It uses high-performance computational codes that combine first-principles discrete particle and continuum descriptions to solve the dynamics of soft matter at the mesoscale, including lengths from nanometers to tens of microns, evolving from nanosecond to minutes. This coarse-grained descriptions of soft matter can capture the essential aspects of the molecular complexity of biomolecules (Angioletti-Uberti, 2017), (Usabiaga et al., 2012) and their hydrodynamic interactions in liquid solvent.

Combining modeling and theoretical analyses, new relations predict the QCM response of discrete biomolecules, either adsorbed (Schofield and Delgado-Buscalioni, 2021) or attached to a linker (e.g., DNA chain) at some distance h over the resonator (Vázquez-Quesada et al., 2020). At low particle density, it is possible to neglect non-linear variations of the impedance with the coverage fraction due to hydrodynamic interactions between analytes. In the case of adsorbed particles (Schofield and Delgado-Buscalioni, 2021), by comparing theory, simulations and experiments, it was found that the linear regime remains valid for surface coverages less than about 10%. In this regime, simple analytical relations (Schofield and Delgado-Buscalioni, 2021) show very good agreement with experiments without any fitting parameters.

This approach will be particularly useful for research on biological samples. It will allow, for the first time, to predict the acoustic behavior of the macromolecular complexes adsorbed on the surface. The individualized coarse grain description of the molecule of interest, either a specific protein, liposomes or viruses, will define all the forces, adhesion, DLVO or elastic, applied to the surface and transferred to the fluid and propagated hydrodynamically to the resonator, creating an extra delayed stress which is sensed by the dissipation signal. By comparing the theoretical predictions to the measured acoustic response, it will be possible to extract information on the deposited mass, height distributions, lateral aggregation or conformational disposition of the biological material being studied. This promises to have a strong impact on the amount of information provided by the QCM with dissipation in protein studies, both in basic research and for future improvements in the design of biosensors and detection schemes (Vázquez-Quesada et al., 2020)*.*

## Atomic Force Microscopy

In the last decades, Atomic Force Microscopy (AFM) has become a common tool to study biological materials (Bednarikova et al., 2020), (Piontek and Roos, 2018), (Dufrêne et al., 2017). Scanning probe microscopes, the family of instruments to which AFM belongs, were developed in the eighties, primarily as surface characterization instruments in solid material science (Binnig and Rohrer, 1999). Since then, their use has increased dramatically and, AFM in particular, has become one of the most powerful tools in biology, materials science, and nanotechnology. Its operating principle is based on sample scanning with a tip mounted at the end of a flexible cantilever. The tip probes the sample surface by monitoring the attractive or repulsive forces between the tip and the surface. The bending of the soft and flexible cantilever obeys Hooke’s law, F = kx, where F is the force on the cantilever, k its spring constant, and x its deflection. As the spring constant of the cantilever is known, either provided by the manufacturer or measured experimentally, this relationship allows obtaining the tip–sample interaction force at different X,Y positions of the scanned surface.

Typically, the cantilever deflection is measured by detecting the position of a laser beam reflected off its backside on a position-sensitive photodetector. There are two main ways that the tip can scan the surface: a static (contact mode) and a dynamic (non-contact or tapping mode), where the cantilever is vibrated. Both modes can operate with the sample surface exposed to air or immersed in a liquid solution, providing the opportunity to study the evolution of surfaces in real-time. In contact mode, the tip touches the sample all along the data acquisition process, whereas in the dynamic mode, the cantilever is induced to oscillate at its resonance frequency at a selected amplitude. The tapping mode is frequently selected to image soft biological materials.

**FIGURE 6:**
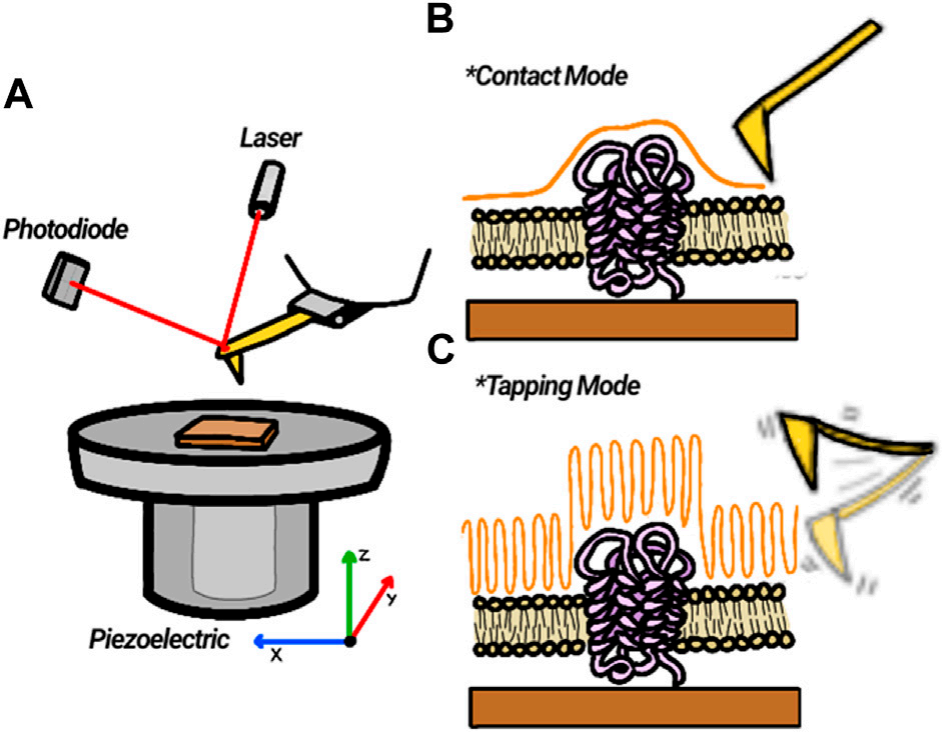
Description of AFM principle. **(A)** A laser reflected from the back of a cantilever impinges on a photodiode and reports on the vertical displacement of the tip as it scans the Surface. **(B)** In contact mode, the tip touches the sample and deflects vertically following surface topography. **(C)** In tapping mode, a tip oscillating at or near its resonant frequency scans over the surface while maintaining its amplitude constant. The piezo vertical displacements reflect the surface topography.

The position of the sample or the tip are controlled using piezoelectric materials that can be moved with very high precision in the nanometer range in all three coordinates X, Y, and Z, allowing for a very fine control of the tip sample distance. Images can be obtained either maintaining the force or the tip oscillating amplitude constant, or maintaining the tip at a fixed distance (constant height mode). At constant force or amplitude mode, the “setpoint”, dependent on the tip-sample interaction, is selected to be kept constant as the tip is scanned over the surface. A feedback loop readjusts the tip-surface distance at each sampling point to cancel any deviations from this “setpoint” caused by surface height changes. The representation of this height modulation (vertical displacement) at each X, Y position is then used to construct a 3D representation of the topography of the surface (Figure 6). Different information about the sample can be obtained during the scanning process, depending on the scanning mode and the information registered. This allows using the microscope as a platform to map other material properties during the acquisition of the topographical image. Local dynamic mechanical properties of the sample can also be studied (Krieg et al., 2019). It is also possible to use the AFM tip as a force sensor to detect interaction forces between a modified tip and material deposited on the surface. Either a sharp tip or a colloidal probe attached to a tipless cantilever can be modified with the desired molecules to test their interaction with molecules found in the surface (Mark et al., 2019), (Yang et al., 2020) More recent instrumental developments have allowed combining information with local measurement of the infrared signal (Dazzi and Prater, 2017), (Mathurin et al., 2020) and increasing the speed to complete images in seconds to follow fast occurring processes (Uchihashi and Scheuring, 2018), (Jiao et al., 2021), (Ando et al., 2014).

Sample preparation for the AFM is of utmost importance. As the technique is strongly sensitive to height differences, it is essential that the material object of study is deposited on top of very flat surfaces. Two frequently used substrates are atomically flat minerals such as mica and HOPG. They are available commercially through different manufacturers that provide materials for AFM analysis. Mica is a clay mineral extensively used in microscopy. Freshly cleaved mica provides a hydrophilic, clean, and atomically flat surface, well suited for depositing SLB by the vesicle fusion method. Highly Oriented Pyrolytic Graphite (HOPG) is a type of pure, highly laminar graphite. The surface is very flat, and, in contrast to mica, it is very hydrophobic. Although the surface of the gold or SiO_2_ evaporated over the quartz crystals is not as flat as mica, selected manufacturers provide surfaces whose roughness remains within a few nanometers, allowing scanning its modified surface with the AFM. Surface characterization and material distribution can be done on the same surface that has been used in the QCM experiment. The area of the surface that is scanned with the AFM is very small, typically between one to one hundred square microns. Even if several images of the same sample are acquired, one cm^2^ of surface covered with, at the most, a monolayer of the material is enough, meaning that the amount of material required for imaging is also very small, which is always convenient when dealing with valuable biological samples.

## Application of QCM and AFM to the study of supported lipid bilayers

In order to illustrate how these two techniques contribute to the study of SLBs of different types, we will describe how they can aid in characterizing different stages of their preparation.

## Formation of the supported lipid bilayer on solid supports

### Vesicle fusion

A first step for the preparation of a supported lipid bilayer using the vesicle fusion method requires a confirmation of the homogeneity of the sample. One frequently accessible approach is to introduce a fluorescent label into the lipids and use fluorescent microscopy techniques to confirm that the vesicles have fused forming a supported lipid bilayer (Kiessling et al., 2017), (Lei and MacDonald, 2003). This technique does not provide enough spatial resolution to quantify the amount of coverage nor to assess and optimize the conditions at which the vesicles of the lipid/protein composition of interest adsorb on the substrate.

QCM follows in real time vesicle adsorption and rupture, estimating also the amount of lipid covering the surface (Reimhult et al., 2009), (Cho et al., 2010). This might be very useful if vesicles with different lipid mixtures are used (Richter and Brisson, 2005) and there is need to optimize the ionic strength, pH, temperature or lipid composition appropriate for vesicle fusion. It allows, for example, determining the effect of buffer salt concentration on the kinetics of the vesicle fusion (Andrews et al., 2021), or to follow the formation of bilayers of a particularly lipid composition (Márquez and Vélez, 2017). Lipids purified from bacteria, *E. coli* for example, can be difficult to fuse. Being able to follow the process allowed optimizing a protocol based on the partial destabilization of the liposomes by the presence of detergent to favor vesicle fusion. As QCM experiments can be carried out under a continuous flow of solution inside the chamber where the crystal is located (illustrated in Figure 4), thorough rinsing of the surface enough to eliminate residuals amount of detergent can be done *in situ*.

When the objective is to prepare SLBs containing proteins, the vesicle fusion of proteoliposomes might present difficulties and might need to be customized for the proteins of interest. QCM, for example, has been used to study how the protein content of aquaporin in the liposomes affects their fusion to the surface (Li et al., 2013). The preparation of tethered bilayers can also be followed properly using the two described techniques (Dorvel et al., 2007).

### Surface coverage and lipid membrane properties

**FIGURE 7:**
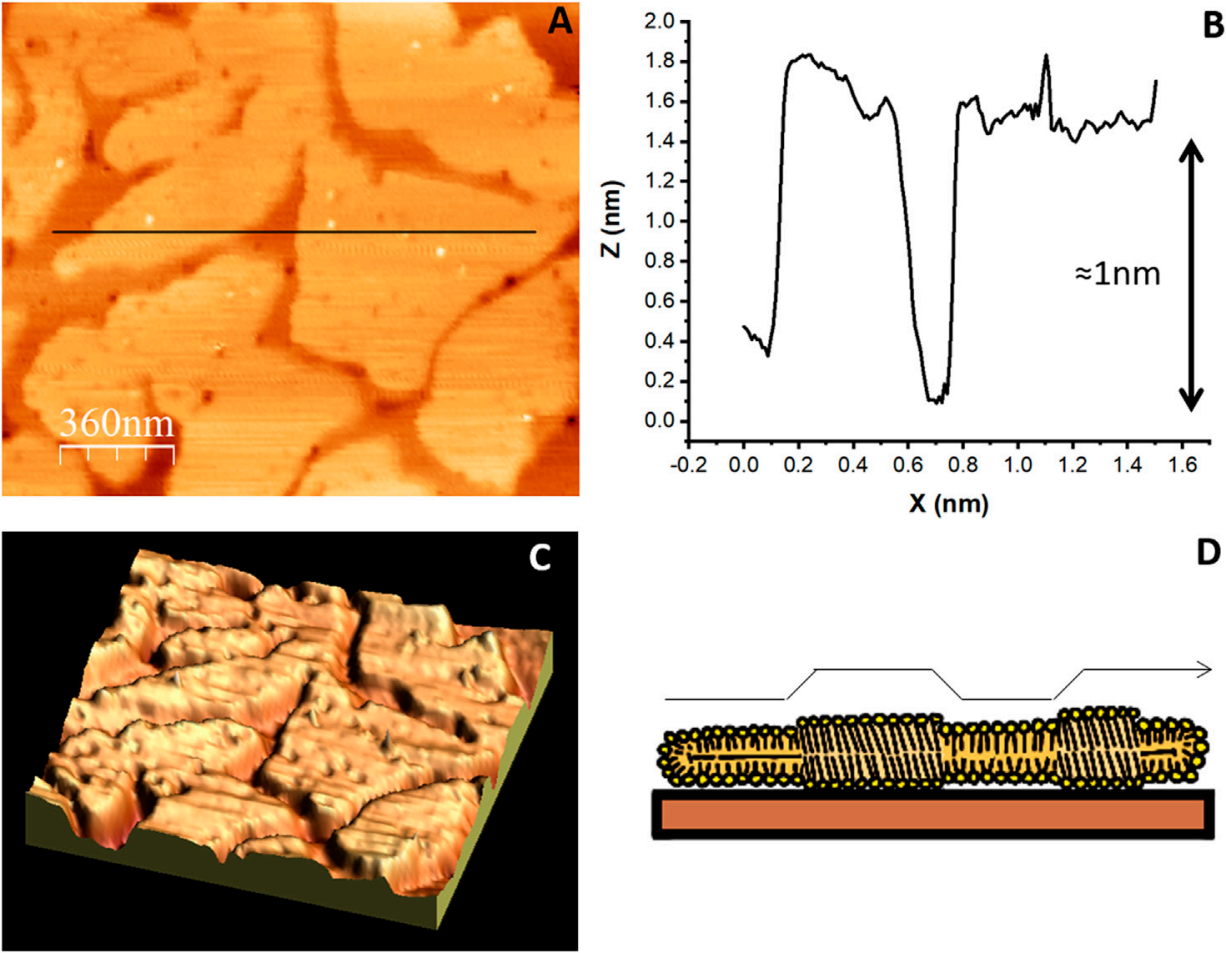
AFM topographic image of an SLB made of a mixture of lipids. **(A)** SLB composed of DPPC 45% DLPC 18% B sitosterol 8% PIP 24% that segregate into liquid ordered and liquid disordered domains (Velez et al., unpublished results). The colors represent differences in height: lighter regions protrude from the surface more than darker regions **(B)** The profile under the black line in **(A)** shows the 1 nm height of the ordered domains. **(C)** Three dimensional representation of the image in **(A)**. **(D)** illustration of the lipid arrangement originating the height difference between ordered and disordered domains.

The changes in Δf detected in the QCM as the SLB is formed reflects the amount of material deposited. Usually, depending on the type of lipid, Δf of a value close to −25 Hz and a ΔD close to zero (Cho et al., 2010) correspond to the formation of a homogeneous bilayer on a 5 MHz quartz resonator. Although this can be taken as good evidence of the formation of a SLB covering the surface, it might be also of interest to visualize the surface with AFM, which allows obtaining a topographical image with nanometer lateral resolution. The surface available for AFM analysis is rather small, within 1–100 μm^2^, depending on the size of the piezo provided by the manufacturer, but the resolution that can be achieved, in the nanometer range, can clearly assess the homogeneity and smoothness of the lipid bilayer. The AFM, as described above, is extremely sensitive to height measurements. It can detect any small holes or imperfections in the bilayer and can also detect with great clarity lateral lipid segregation of the lipids forming the bilayer (El Kirat et al., 2010), (Morandat et al., 2013), (Goksu et al., 2009). Figure 7 shows how an AFM image can identify different lipid domains in a bilayer that differ in height. Moreover, as the AFM tip is able to sense and apply forces with pN sensitivity, it is also an excellent tool to gather information about molecular interactions at the single molecule level through what is known as force spectroscopy (FS), when the tip is approached to the sample in a controlled manner until the bilayer underneath ruptures (Dufrêne et al., 1999), (Garcia-Manyes and Sanz, 2010). This rupture force is then a measure of the mechanical stability of the bilayer and permits to associate the effect of lipid head (Garcia-Manyes et al., 2010), and cation type (Redondo-Morata et al., 2014) on the mechanical stability of the bilayer.

Another aspect of SLBs that is of great interest is the study of the phase transition temperature of the constituting lipids and temperature dependent lipid segregation. Both AFM and QCM with dissipation can be coupled to temperature control units that allow performing experiments while maintaining the substrate immersed in solution. In both cases, the sensitivity of the measurements requires equipment adaptations that provide enough stability, and not all commercially available equipments have this option. Furthermore, special care should be taken while performing and interpreting the QCM data as changes in the viscosity and density of the solution due to temperature should be taken into consideration.

AFM can visualize and follow domain formation as a function of temperature, with the characteristic nanometer lateral resolution of the technique [(Alessandrini and Facci, 2014), (Redondo-Morata et al., 2020)] therefore extending the spatial resolution attainable using fluorescence microscopy and providing also the possibility of probing the local mechanical properties of the lipid domains.

The development of high speed AFM (Uchihashi and Scheuring, 2018), which can take images every few seconds, as compared to several minutes of a standard AFM, will certainly improve the time resolution required to obtain a more thorough kinetic description of lipid phase transitions and local perturbations due to peptide interactions (Jiao et al., 2021).

QCM with dissipation has also been used to directly detect the gel−fluid phase transition of a supported lipid bilayer (Jing et al., 2014), (Redondo-Morata et al., 2020). One recent approach consisted in comparing the frequency response of a bare and a bilayer-coated QCM crystal during linear temperature variation. Phase transition results in a change of the resonance frequency that coincides directly with the accompanied change in bilayer thickness detected by ellipsometry. The phase transition was detected based on the temperature-induced viscosity changes of the ambient medium in the immediate environment of the bilayer. The authors describe the great potential of the approach for sensitive detection of structural and/or compositional changes of the bilayer (Wargenau and Tufenkji, 2014). The sensitivity of the technique to the mass density of supported membranes is well-suited also to examine surface adsorption and membrane disruption phenomena. The detection of the phase transition in the presence of phenolic compounds manifested noticeable alterations in their gel−fluid phase transitions. This confirmed that QCM can detect compound-specific lipid interactions by detecting small variations in a SLB’s main transition temperature (≪1°C) (Wargenau et al., 2018).

## Protein and peptide interaction with SLBs

### Spatial arrangement of peptides and proteins on SLBs

Another process in which both techniques have provided relevant information is the study of how certain peptides or membrane associated proteins interact with lipid bilayers. There are different aspects of this problem that can be addressed. On one hand, it might be interesting to follow how the peptide or protein distribute on a lipid surface, whether they aggregate forming domains, whether these are associated or determined by specific segregated lipids on the surface. On the other hand, it might be of interest to describe any membrane modifications or remodeling due to the presence of the peptides or proteins. These morphological characterizations can best be described using AFM, because in many instances the nanometer resolution can provide a very fine description of pore forming peptides. An example of the combined used of both techniques to learn about antimicrobial peptides (AMPs) interaction with membranes is provided by the work in Camesano’s group. QCM with dissipation analysis allowed differentiating dynamics of peptide–membrane interactions and the rates at which different antimicrobial peptides interacted with the membrane and, in some cases, induced lipid removal (Wang et al., 2015). Later studies using AFM were able to complement the study by describing the time evolution and extent of lipid removal from the membrane by documenting the amount and stability of defects in the membrane generated by different peptide concentrations (Swana et al., 2021). The convenience of using QCM to analyze antimicrobial peptide interaction with membranes has motivated exploring different approaches to interpret the data in order to extract the maximum information (McCubbin et al., 2011). Frequency–dissipation plots (Δf- ΔD plots) are used to ascertain the mechanism of action of the AMP. The aim is to gather enough information to build a database of fingerprints that define antimicrobial peptide membrane interactions to help in the future development of antibiotics (McCubbin et al., 2011). It is however important to realize that one of the limitations of the acoustic method is that these viscoelastic fingerprints are also sensitive to the membrane model system, multilamellar membrane stacks or unilamellar liposomes bilayer membrane and this should be considered in studies of the mechanism of action of AMPs (Pandidan and Mechler, 2020).

The study of the interaction of β-amyloids with model membranes is another typical example that illustrates the contribution of AFM and QCM with dissipation to the study of biomimetic systems. Work from S. Jarvis groups clearly demonstrated that the mode and rate of the interaction of β-amyloid monomer with lipid bilayers are strongly dependent on lipid composition, phase state and cholesterol content (Sheikh et al., 2012). Work coming from Nam-Joon Cho’s lab demonstrated, using QCM with dissipation and fluorescence microscopy, that membrane thickness influences the membrane morphological response triggered upon Aβ amyloid adsorption (Meker et al., 2018).

High speed AFM also offers a wonderful opportunity to document not only the morphological changes induced by the peptides or proteins on the membrane, but also to record their dynamics occurring with a time resolution of seconds (Feuillie et al., 2020), (Jiao et al., 2021), (Heath et al., 2021).

**FIGURE 8:**
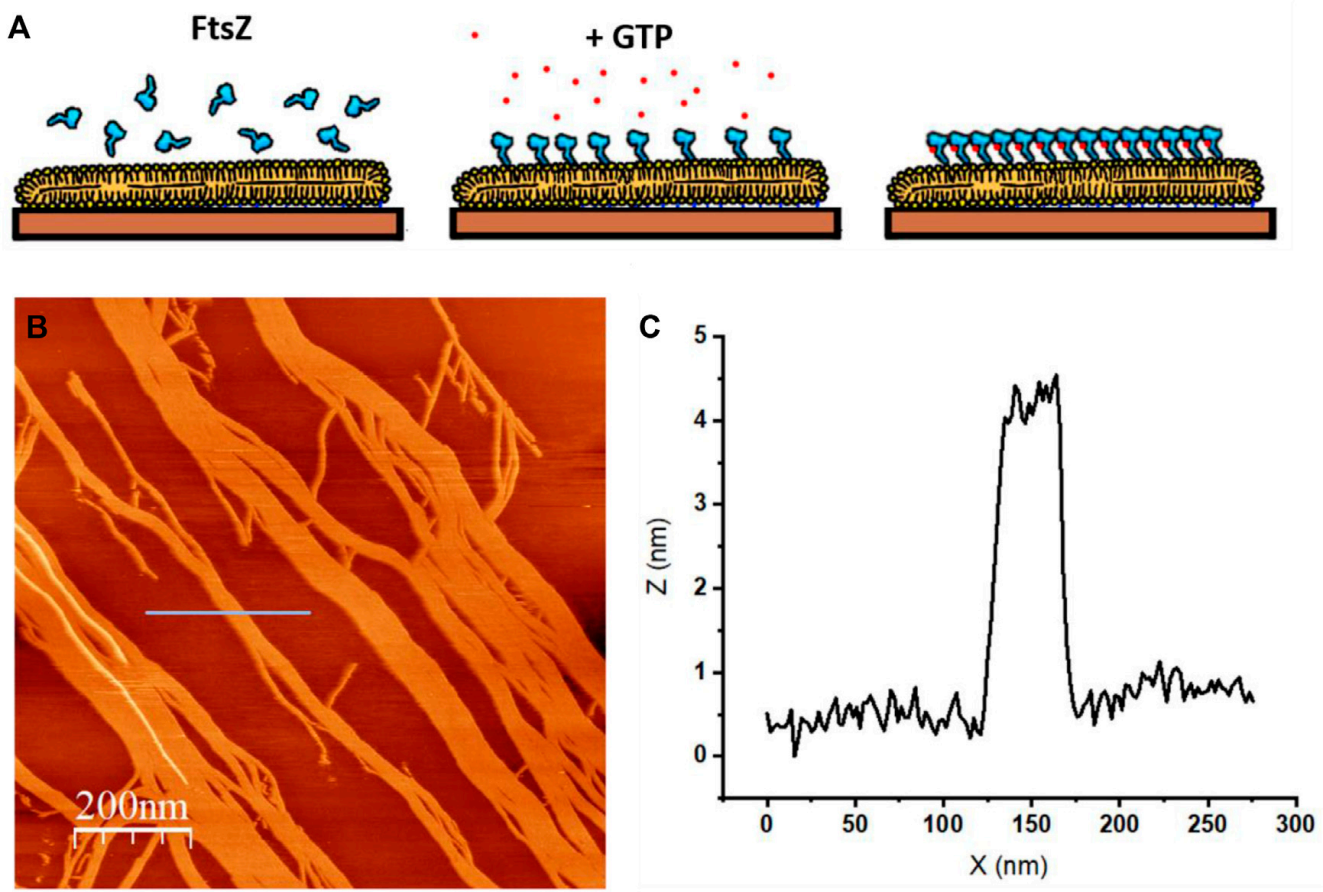
AFM topographic image of protein seggregation in an SLB. **(A)** The protein is bacterial cytoskeletal protein FtsZ covalently attached to a lipid bilayer as described in (Márquez et al., 2019). FtsZ protein forms filaments in the presence of GTP **(B)** Topographic image that shows the filaments formed on top of the SLB upon GTP addition. The colors represent differences in height: lighter regions protrude from the surface more than darker regions (unpublished image from Velez’s lab.) **(C)** Profile under the blue line in **(B)** that shows the height of the filaments.

It is sometimes of interest to consider how membrane associated proteins interact and distribute on a lipid surface and whether this distribution is associated or not to lipid segregation (Seeger et al., 2009). The high spatial resolution of the AFM is well suited to answer this question, as it can clearly distinguish between lipid domains (Figure 8) and proteins, protruding from the membrane, either inserted or adsorbed on the surface (Faiß et al., 2008), (Márquez et al., 2019). The pH and ionic content of the imaging solution can vary, therefore allowing to interrogate their effect on lipid-protein interactions without the need of introducing any external label.

QCM, on the other hand, allows quantifying the kinetics and thermodynamics of biomolecular interactions between soluble ligands and a surface (Höök and Kasemo, 2006). One example of proteins whose membrane affinity has been characterized using QCM are annexins, a family of cellular proteins that reversibly bind to cellular membranes in a Ca^2+^—dependent manner. Rate and affinity constants of annexin A1 binding to phosphatidylserine-containing layers as a function of the calcium ion concentration in solution and the cholesterol content within the outer leaflet of the solid-supported bilayer were measured (Kastl et al., 2002), (Gerke and Moss, 2002), (Ross et al., 2003). Further studies including also AFM imaging and Monte Carlo simulations allowed a better understanding of the Ca^2+^ concentration conditions at which the process became irreversible (Kastl et al., 2006).

### Measuring biomolecular interactions

The configuration of the acoustic sensor is also appropriate for describing the interaction between soluble ligands and membrane receptors or membrane associated proteins, which can be either inserted or associated in the lipid membrane covering the quartz crystal surface exposing their active region to the solution. Upon binding of the ligand or protein, the change of mass on the crystal surface is detected as a frequency decrease allowing for detection of the binding kinetics and affinities. Two systems in which this type of setup has been used is for studying IgG binding kinetics to oligo B protein A domains immobilized on lipid layers (Mitomo et al., 2007) and for studying the binding of F-actin filaments to ponticulin included in supported bilayers (Barfoot et al., 2008). This represents a good example of the limitations of using QCM with dissipation to associate frequency changes as mass when the morphology of the bound proteins cannot be characterized as an homogeneous layer. The authors used an auxiliary technique, Surface Plasmon Resonance (SPR) to measure the amount of material deposited on the surface to estimate the affinity constants, as the ∆f and ∆D values measured did not follow the expected results. The authors modeled the results as if the filaments at increasing concentration adsorbed as an homogeneous viscoelastic layer, which is not necessarily the case (Barfoot et al., 2008). Other reports looking at the binding of FtsZ filaments on ZipA at a lipid surface have also detected an increase of the dissipation ∆D at increasing filament concentration on the surface (Sobrinos-Sanguino et al., 2019). These results clearly illustrate that advancements in the theoretical analysis of QCM are needed in the field to interpret results of complex systems. Associating the increased dissipative signal with protein rearrangement and morphology changes at the interface will provide more reliable and thorough information about molecular configurations at the lipid interface.

### Intrinsically disordered proteins near surfaces

One last potential application of both QCM with dissipation and AFM worth mentioning is the study of intrinsically disordered proteins (IDPs) near surfaces. IDPs, because of their lack of stable well defined conformation, are difficult to study using well established structural techniques, in spite of their increasingly documented importance in biological events occurring near the membrane surfaces (Cornish et al., 2020), (Das and Eliezer, 2019). QCM senses the hydrodynamics at the interface between the liquid and the crystal. Therefore, conformational changes that either extend or collapse a protein in the vicinity of the surface can be monitored (Mateos-Gil et al., 2016), (Shur et al., 2011). It is likely that the development of theoretical analysis that more closely describe these events in non-homogeneous layers will facilitate experiment interpretation and design and contribute to extend this type of analysis to other IDPs near lipid membranes.

High speed AFM has also been recently used to explore IDPs deposited on mica (Kodera et al., 2021). This analysis was able to identify constantly folded and constantly disordered regions and disorder-to-order transitions in the molecule.

## Summary and outlook

We have presented the fundamental operation principles of QCM with dissipation and AFM, two techniques that in the past years have been extensively and fruitfully used to characterize supported lipid bilayers. Both techniques originated in fields different from biology to characterize interfaces, but as they can both be operated with the surface immersed in solution, at some point they became of great help to study biological surfaces. In the last couple of decades, they have both settled down as powerful tools in biophysical studies. Enough information about their operating principles is provided in order to illustrate their potential and their limitations. In the case of the QCM, it is clear that on-going theoretical developments will facilitate further and more extensive use in basic research. The more we understand the hydrodynamic behavior of soft biological material on an oscillating surface, the more information we will extract from the behavior of protein assemblies originating it. Data interpretation is strongly theory dependent, which has motivated developing equipment coupled to other techniques such as ellipsometry (Richter and Brisson, 2005), (Van Der Meulen et al., 2014) in order to obtain independent mass measurements. There are commercially available equipments combining both techniques. Higher frequency resonators offering increased sensitivity are also available. The versatility of the device is also illustrated by the fact that the gold surface of the oscillating crystal can be used as a working electrode, allowing to follow changes in the mass deposited on the surface as a function of the voltage of the electrochemically active surface (Ji et al., 2021).

Although we have mostly focused on applications related to characterizing SLBs, there are other biological applications for QCM with dissipation that include biomedical (Casero et al., 2010), (Bragazzi et al., 2015), (Ding et al., 2020), (Farooq et al., 2018), environmental sensing (Kumar et al., 2020), (Mi et al., 2021), or following cell attachment (Fathi et al., 2018), (Kilic and Kok, 2019), (Chen et al., 2018).

The use of Atomic Force Microscopy is probably more widespread as a standard tool to characterize SLBs than the acoustic sensor. Its contribution to the field is now well accepted. However, it is also undergoing frequent improvements and being combined with other complementary techniques that make it even more powerful. One good example is its combination with fluorescence microscopy. Although this requires the use of incorporating fluorescent labels to the studied systems, the capacity to obtain structural information from the micro to the nanometer scales is very promising. It is also expected that advances in combining superresolution fluorescence imaging with AFM will be very fruitful (Wang et al., 2020).

In brief, we have presented two complementary surface characterization techniques that, in combination, provide kinetic, thermodynamic and structural information at the nanometer scale of events taking place at the surface of supported lipid bilayers. Both techniques, although well established in biological research, are still undergoing continuous developments that will most likely strengthen and increase their impact. We believe that a better understanding of their basic fundamental principles will contribute to extend their use by aiding to develop a critical evaluation of their strengths and limitations.
